# Private Benevolent Societies and the Census

**Published:** 1890-07

**Authors:** 


### PRIVATE BENEVOLENT SOCIETIES AND THE CENSUS.

No organizations in the United States have multiplied more rapidly in the past ten years than the sick-benefit, funeral-aid, death-benefit, and other kindred societies.

As they are generally confined to those who are in the humbler walks of life, the good they have done is incalculable, carrying substantial aid to thousands of stricken families, and inspiring those who are fortunate enough in being members with a courage which might not exist in their hearts without them.

The members of these organizations will be glad to learn that Hon. ROBERT P. PORTER, Superintendent of the Eleventh Census, will endeavor to secure the statistics of the noble work these associations are doing, and it is safe to say that no other branch of the census will be more interesting.

The business of gathering the data has been placed in charge of Mr. CHARLES A. JENNEY, special agent of the insurance division, 58 William street, New York city, and all associations throughout the United States, whether incorporated or private, should assist by sending to him the address of their principal officers.

ANCIENT BREAD.—Crackers are the oldest form of bread known. In the ruins of the Swiss buildings which belonged to the neothelic age, fragments of unfermented cakes have been discovered, which were not very unlike our modern crackers.

THE "sulphur-cure" for diphtheria has been extensively published by the press, and has some medical indorsement. Sulphur may be good for diphtheria, but it certainly would not be safe to inhale the fumes of burning sulphur. A deadly agent—sulphurous acid gas—is thus generated, the inhalation of which would almost inevitably prove fatal.

